# The impact of pineapple consumption on cervical ripening and labor outcomes: A Nationwide Retrospective Cohort Study among pregnant women in Nigeria

**DOI:** 10.1097/MD.0000000000048335

**Published:** 2026-04-17

**Authors:** Chukwuka Elendu, Dependable C. Amaechi, Tochi C. Elendu, Emmanuel C. Amaechi, Ijeoma D. Elendu, Joy Onomon, Egbunu Emmanuel, Peace Mordi, Chinonso B. Okoro

**Affiliations:** a Department of Medicine, Federal University Teaching Hospital, Owerri, Nigeria; b Department of Medicine, Igbinedion University, Okada, Nigeria; c Department of Medicine, Imo State University, Owerri, Nigeria; d Department of Medicine, Madonna University, Elele, Nigeria; e Department of Medicine, Royal United Hospital, Bath, England; f Department of Medicine, Federal Medical Centre, Bida, Nigeria; g Department of Medicine, Gannan Medical University, Ganzhou, Jiangxi, China; h Department of Medicine, University of Nigeria Teaching Hospital, Ituku-Ozalla, Nigeria.

**Keywords:** cervical ripening, labor outcomes, maternal health, pineapple consumption, retrospective cohort study

## Abstract

Cervical ripening, a vital precursor to labor, involves complex biochemical and structural changes in the cervix. While medical interventions like prostaglandins and oxytocin are commonly used, dietary factors such as pineapple, containing bromelain – a proteolytic enzyme hypothesized to aid cervical tissue remodeling – have gained attention. This study evaluates the impact of pineapple consumption on cervical ripening and labor outcomes among pregnant women in Nigeria. This nationwide retrospective cohort study included pregnant women aged 18 to 45 years who delivered between January 1, 2018, and December 31, 2023. Participants were categorized into 2 cohorts: those reporting regular 3rd-trimester pineapple consumption (exposed) and those reporting no consumption (unexposed). Data were collected from medical records and structured questionnaires. A total sample size of 2400 participants (1200 exposed and 1200 unexposed) was included based on predefined power calculations. Primary outcomes included cervical ripening (Bishop score), labor duration, and mode of delivery, while secondary outcomes assessed neonatal health. Statistical analysis employed multivariable regression models to adjust for confounding factors such as maternal age, parity, body mass index, gestational age, and socioeconomic status. Linear regression was used for continuous outcomes and logistic regression for binary outcomes. Analyses were performed using Stata Statistical Software: Release 17 (StataCorp LLC, College Station). In multivariable cohort analyses, pineapple consumption was associated with significantly improved cervical ripening (adjusted mean difference 0.8, 95% CI 0.5–1.1, *P* < .001). Labor duration was shorter among the exposed group (adjusted mean difference −1.7 hours, 95% CI −2.1 to −1.3, *P* < .001). Women in the exposed cohort had higher odds of spontaneous vaginal delivery (adjusted odds ratio [aOR] 1.8, 95% CI 1.4–2.3, *P* < .001), with lower cesarean delivery rates. Neonatal outcomes, including birth weight, Apgar scores, and neonatal intensive care unit admissions, showed no significant differences between groups after adjustment. Dose–response analysis demonstrated that higher pineapple consumption was independently associated with shorter labor duration and increased likelihood of vaginal delivery. Third-trimester pineapple consumption was associated with improved cervical ripening, shorter labor duration, and higher rates of spontaneous vaginal delivery without adverse neonatal outcomes. These findings support further investigation of dietary influences on labor outcomes.

## 1. Introduction

Cervical ripening is a critical physiological process preceding the onset of labor, characterized by progressive biochemical, structural, and functional changes within the cervical extracellular matrix. These changes, which include collagen remodeling, increased tissue hydration, and altered inflammatory signaling, facilitate cervical softening and dilation necessary for vaginal delivery. In clinical practice, pharmacologic agents such as prostaglandins and oxytocin, as well as mechanical methods, are routinely employed to promote cervical ripening and initiate labor when indicated.^[1–3]^ In parallel, increasing attention has been directed toward nonpharmacologic and dietary factors that may influence labor physiology, including their potential effects on labor duration, mode of delivery, and childbirth-related complications, particularly in settings where access to medical interventions may be limited.

Among dietary exposures of interest, pineapple consumption has attracted attention due to its content of bromelain, a proteolytic enzyme with established antiinflammatory and protease activity. Bromelain has been hypothesized to influence connective tissue remodeling through its effects on collagen and extracellular matrix proteins, processes central to cervical ripening.^[4–6]^ Within traditional and anecdotal contexts, pineapple is frequently believed to facilitate labor, with such beliefs often attributed to bromelain-mediated collagen degradation. Beyond cervical changes, this proposed mechanism has been speculated to influence downstream labor outcomes, including the duration of labor, the likelihood of spontaneous vaginal delivery, and the need for obstetric interventions. However, the biological plausibility of these associations remains incompletely understood, and direct clinical evidence linking pineapple consumption to cervical ripening, labor progression, or neonatal outcomes is limited.

Most existing research on bromelain has focused on its pharmacologic properties in inflammation modulation, wound healing, and tissue permeability, rather than its role in parturition or obstetric outcomes.^[7]^ Moreover, experimental studies demonstrating bromelain enzymatic activity have largely been conducted in vitro or using purified extracts, leaving uncertainty regarding its bioavailability, effective dosage, and physiological impact when consumed as part of whole pineapple fruit.^[7,8]^ A limited number of analytical and observational studies examining pineapple or bromelain exposure during pregnancy have reported mixed findings, with some suggesting shorter labor duration or improved cervical favorability, while others report no significant association with labor outcomes or neonatal parameters. These discrepancies may reflect differences in study design, exposure assessment, population characteristics, and outcome definitions.^[4,5]^

Nigeria provides a relevant context in which to examine this association due to its population size and heterogeneity. The country is characterized by substantial regional, cultural, and socioeconomic diversity, accompanied by wide variation in dietary practices and traditional beliefs surrounding pregnancy and childbirth. Pineapple is widely available and commonly consumed across many regions, and in some communities is promoted as a natural means of facilitating labor. In contrast, other cultural settings discourage its intake during pregnancy due to concerns about adverse outcomes. These contrasting practices provide an opportunity to examine associations between pineapple consumption and measurable childbirth outcomes, rather than focusing solely on cultural beliefs.^[6]^

This knowledge gap is especially pertinent given Nigeria maternal health burden. The country accounts for a disproportionate share of global maternal and neonatal mortality, with labor-related complications – such as prolonged labor, obstructed labor, postpartum hemorrhage, and adverse neonatal outcomes – remaining major contributors to morbidity and mortality.^[8–10]^ Understanding factors associated with labor duration and childbirth complications may therefore have important clinical and public health implications.

Although a growing body of literature suggests that diets rich in fruits and vegetables may be associated with improved pregnancy outcomes – such as reduced risks of preeclampsia and gestational diabetes – evidence specific to labor initiation, labor progression, and neonatal outcomes remains sparse and inconsistent.^[5,6]^ Pineapple, as a source of bromelain as well as micronutrients including vitamin C and manganese, has been discussed within this broader nutritional context. However, concerns persist regarding its safety in pregnancy, particularly the possibility of precipitating uterine activity, preterm labor, or adverse neonatal outcomes. These concerns are largely anecdotal and lack robust epidemiologic confirmation, underscoring the need for population-based data.

To date, most studies examining dietary influences on labor and childbirth outcomes have been conducted in high-income settings, limiting their generalizability to low- and middle-income countries.^[11,12]^ A nationwide retrospective cohort study provides an opportunity to evaluate the association between pineapple consumption during pregnancy and key maternal outcomes (including cervical ripening, labor duration, mode of delivery, and childbirth complications) as well as neonatal outcomes, while accounting for regional, demographic, and healthcare-related variability.^[13]^

By systematically examining these associations, the present study aims to generate evidence that may inform clinical counseling, public health guidance, and future mechanistic research. Whether pineapple consumption is associated with favorable labor and neonatal outcomes, neutral effects, or potential risks, clarifying this relationship is essential for evidence-based dietary recommendations during pregnancy in Nigeria and similar settings.^[14–16]^

## 2. Methods

### 2.1. Study design

This nationwide retrospective cohort study investigated the relationship between pineapple consumption during pregnancy and cervical ripening and labor outcomes among Nigerian women. Retrospective cohort methods were chosen to analyze existing data derived from standardized maternal dietary questionnaires and obstetric medical records across multiple healthcare facilities in Nigeria. The primary goal was to evaluate differences in labor outcomes, including cervical ripening, duration of labor, induction-to-delivery intervals, and mode of delivery, between women who reported pineapple consumption and those who did not.

### 2.2. Setting

The study was conducted in public and private healthcare facilities across all 6 geopolitical zones of Nigeria to ensure representative national coverage. Data collection spanned January 1, 2018, to December 31, 2023. Healthcare facilities were selected based on maternity service volume, completeness of obstetric records, and availability of antenatal dietary documentation. Data abstraction was performed retrospectively by trained research assistants and obstetric clinicians using a standardized data extraction protocol across all sites.

### 2.3. Participants

Eligibility criteria included pregnant women aged 18 to 45 years who delivered in participating facilities during the study period. Women with multiple gestations (e.g., twin or higher-order pregnancies), known chronic medical illnesses (including pregestational diabetes mellitus, chronic hypertension, autoimmune disorders), or those receiving long-term medications known to influence uterine contractility or cervical integrity were excluded. Additional exclusion criteria included incomplete medical records, known uterine anomalies, cervical insufficiency, or a history of elective cesarean section unrelated to labor progression.

Participants were stratified into 2 cohorts:

1. women who reported pineapple consumption during pregnancy (exposed group), and
2. women who reported no pineapple consumption during pregnancy (unexposed group).

Exposure data were obtained through structured, interviewer-administered dietary questionnaires, supplemented by antenatal records where available. Questionnaires were completed either during routine antenatal visits or within 48 hours postpartum to minimize recall error. Follow-up was not applicable due to the retrospective design.

### 2.4. Questionnaire design, validity, and reliability

Dietary exposure was assessed using a standardized food-frequency questionnaire adapted from previously validated maternal dietary assessment tools and modified to include pineapple consumption patterns relevant to the Nigerian context. The questionnaire captured frequency, quantity, and timing of pineapple intake. Content validity was assessed by obstetricians and nutrition experts prior to deployment, and internal consistency reliability was confirmed in a pilot sample, yielding a Cronbach alpha of ≥0.80.

### 2.5. Exposure assessment: pineapple consumption

Pineapple consumption was evaluated based on self-reported intake during pregnancy, with primary exposure defined as consumption during the 3rd trimester, the period most relevant to cervical ripening and labor initiation. The questionnaire assessed average frequency (times/wk) and quantity (standard serving size per intake) over the preceding 4 to 8 weeks prior to delivery. Participants reporting sporadic or infrequent intake (<1 serving/wk) were classified separately in sensitivity analyses.

### 2.6. Outcome measures

Primary outcomes included cervical ripening, assessed using the Bishop score recorded at hospital admission for labor, duration of labor, induction-to-delivery interval, and mode of delivery. The Bishop score was obtained from standardized cervical examinations documented by attending obstetricians or midwives, following routine clinical practice. Secondary outcomes included neonatal outcomes such as birth weight, 1- and 5-minutes Apgar scores, and neonatal intensive care unit (NICU) admission.

### 2.7. Data collection procedures

Over the 5-year study period, data were collected retrospectively from hospital delivery registers, antenatal care records, labor ward documentation, and completed questionnaires. Data abstraction was performed by trained personnel using uniform data collection templates, and periodic cross-checks were conducted to ensure consistency across sites.

### 2.8. Study size and sample size calculation

The sample size was calculated to detect a minimum clinically significant difference in Bishop score between exposed and unexposed groups. Using the formula for comparison of 2 independent means, assuming a 2-sided α of 0.05, power of 80%, an expected mean difference (effect size) of 0.5 points in Bishop score between groups was assumed based on prior obstetric literature, with an estimated standard deviation (SD) of 2.0 points. Under these assumptions, the minimum required sample size was calculated as 1200 women per group, yielding a total sample size of 2400 participants. An additional 10% was incorporated to account for incomplete records or missing exposure data, ensuring adequate statistical power for primary analyses.

### 2.9. Bias

Efforts to reduce recall bias included restricting dietary recall to the late antenatal or immediate postpartum period and cross-verifying self-reported pineapple intake with antenatal documentation where available. Selection bias was minimized by including facilities across diverse geographic and socioeconomic settings. Potential confounders were prespecified and adjusted for in multivariable models.

### 2.10. Statistical methods

Statistical analyses compared cervical ripening scores, labor duration, and delivery outcomes between exposure groups using multivariable linear regression for continuous variables and logistic regression for binary outcomes, adjusting for maternal age, parity, gestational age, body mass index (BMI), medical comorbidities, socioeconomic status, and dietary patterns. Subgroup analyses were conducted by parity and maternal age. Missing data were handled using multiple imputation, and sensitivity analyses tested alternative exposure definitions and outcome thresholds.

### 2.11. Ethical considerations

Ethical approval was obtained from the Health Research Ethics Committee of Federal University Teaching Hospital Owerri, Nigeria, in accordance with the guidelines of the National Health Research Ethics Committee of Nigeria, with approval number: FUTHO/HREC/2023/041. Institutional permissions were obtained from all participating facilities. All data were anonymized prior to analysis, and informed consent requirements were waived due to the retrospective nature of the study.

## 3. Results

### 3.1. Participants

**Figure 1.**
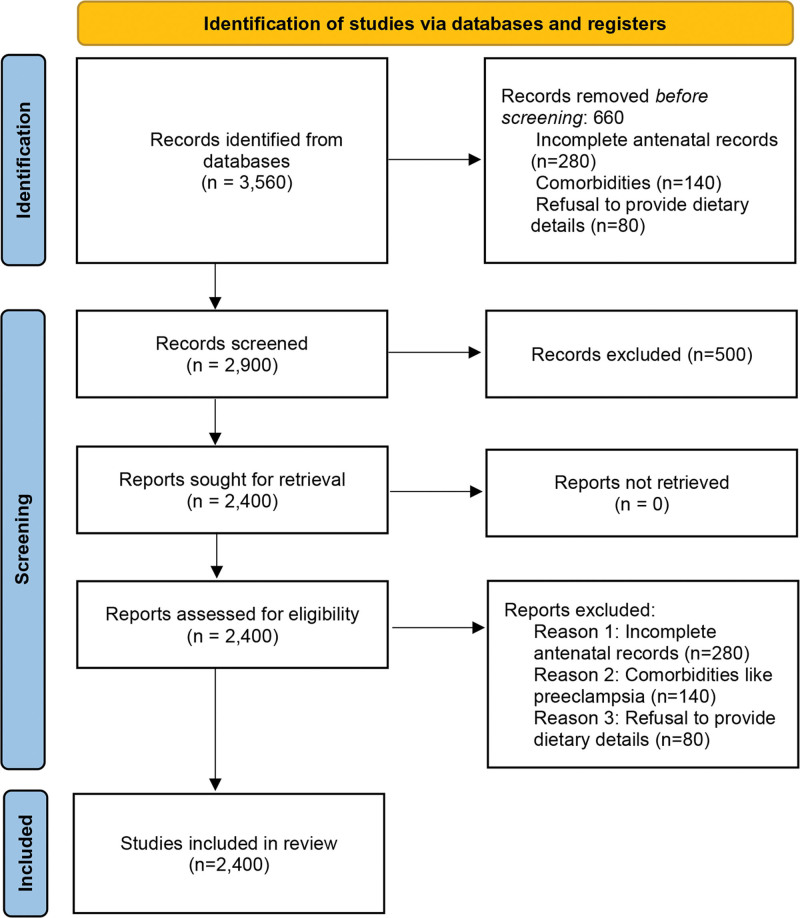
Participant flow diagram. This structured representation outlines the flow of participants from initial identification to final analysis, reflecting the exclusions and grouping for the study. Source: Authors’ Creations.

Out of 3560 pregnant women initially identified as potentially eligible for the study, 2900 met the inclusion criteria. Two hundred participants were included in the final analysis after excluding 500 women due to incomplete records or contraindicating medical conditions. The exposed group (pineapple consumers) comprised 1200 women, while the unexposed group (nonconsumers) included 1200 women. Reasons for nonparticipation included incomplete antenatal records (n = 280), exclusion due to comorbidities such as preeclampsia or gestational diabetes (n = 140), and refusal to provide dietary details (n = 80). The final sample size adhered to the calculated power requirements, ensuring sufficient representation for both groups. The participant flow is summarized in (Fig. 1), detailing the selection process from initial eligibility screening to final analysis.

### 3.2. Descriptive data

Baseline characteristics of the study participants are presented in (Table 1). The median maternal age was 30 years (IQR 27–34), with 55% of the participants aged 26 to 35. The exposed group reported a higher prevalence of nulliparity (40%) than the unexposed group (35%). Key demographic and clinical variables were balanced across the 2 groups after adjustments: gestational age at delivery: mean 39.2 weeks (SD 1.1) in both groups; BMI: mean 26.5 kg/m^2^ (SD 2.8) in the exposed group and 26.3 kg/m^2^ (SD 2.9) in the unexposed group; socioeconomic status: equal distribution between low-income, middle-income, and high-income groups. Missing data accounted for <5% of all variables and were imputed using multiple imputation techniques. Pineapple consumption was categorized based on weekly intake frequency derived from dietary records: low consumption (≤2 servings/wk), moderate consumption (3–5 servings/wk), and high consumption (≥6 servings/wk).

**Table 1:** Baseline characteristics of study participants.

Variable	Exposed group (n = 1200)	Unexposed group (n = 1200)	*P* value
Maternal age (yr)			
Median (IQR)	30 (27–34)	30 (27–34)	.82
Age categories, n (%)			
≤25	240 (20%)	240 (20%)	
26–35	660 (55%)	660 (55%)	
>35	300 (25%)	300 (25%)	
Parity, n (%)			
Nulliparous	480 (40%)	420 (35%)	.04
Multiparous	720 (60%)	780 (65%)	
Gestational age at delivery (wk)			
Mean (SD)	39.2 (1.1)	39.2 (1.1)	.98
Body mass index (kg/m^2^)			
Mean (SD)	26.5 (2.8)	26.3 (2.9)	.33
Socioeconomic status, n (%)			
Low-income	300 (25%)	300 (25%)	.87
Middle-income	480 (40%)	480 (40%)	
High-income	420 (35%)	420 (35%)	
*Pineapple consumption category, n (%)			
Low (≤2 servings/wk)	300 (25%)	N/A	
Moderate (3–5 servings/wk)	600 (50%)	N/A	
High (≥6 servings/wk)	300 (25%)	N/A	

* Pineapple consumption categories defined according to weekly intake frequency derived from dietary records.

Initial comparisons between exposure groups were explored using independent *t* tests or chi-square tests as appropriate; however, these analyses were considered exploratory and were followed by multivariable regression models to account for confounding and reflect the retrospective cohort design.

### 3.3. Outcome data

**Figure 2.**
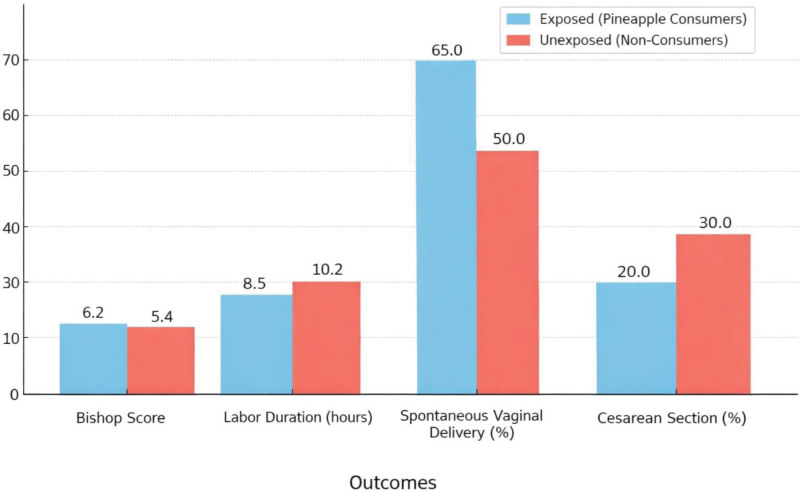
Bar chart comparing primary obstetric outcomes between exposed (pineapple consumers) and unexposed (nonconsumers) cohorts, demonstrating higher mean Bishop scores, shorter labor duration, increased spontaneous vaginal delivery rates, and reduced cesarean section rates in the exposed group.

Primary and secondary outcomes are summarized in Table 2. First,cervical ripening: the mean Bishop score at admission was significantly higher in the exposed group (6.2 ± 1.3) compared to the unexposed group (5.4 ± 1.5), *P* < .001. Second, labor duration: the average duration of labor was shorter in the exposed group (8.5 ± 2.1 hours) compared to the unexposed group (10.2 ± 2.4 hours), *P* < .001. Third, mode of delivery: spontaneous vaginal delivery occurred in 65% of the exposed group and 50% of the unexposed group, *P* = .002. Cesarean section rates were lower in the exposed group (20%) compared to the unexposed group (30%), *P* = .005 (see Fig. 2). Secondary outcomes showed no significant differences in neonatal birth weight, Apgar scores, or NICU admissions between the 2 groups.

**Table 2:** Primary and secondary outcomes comparing pineapple consumers and nonconsumers.

Outcome	Exposed group (pineapple consumers)	Unexposed group (nonconsumers)	*P* value
Primary outcomes			
Mean Bishop score (±SD)	6.2 ± 1.3	5.4 ± 1.5	<.001
Labor duration (h, ±SD)	8.5 ± 2.1	10.2 ± 2.4	<.001
Spontaneous vaginal delivery (%)	65%	50%	.002
Cesarean section (%)	20%	30%	.005
Secondary outcomes			
Neonatal birth weight (kg, ±SD)	3.2 ± 0.4	3.1 ± 0.5	.07
Apgar score (5 min, median)	9 (IQR 8–9)	9 (IQR 8–9)	.83
NICU admissions (%)	8%	9%	.65

Key Insights.

• Primary outcomes: Pineapple consumption was associated with significantly improved cervical ripening, shorter labor durations, and higher rates of spontaneous vaginal delivery (see Figure 2).

• Secondary outcomes: No significant differences were observed in neonatal birth weight, Apgar scores, or NICU admission rates between the 2 groups.

NICU = neonatal intensive care unit, SD = standard deviation.

Source: Authors’ Creations.

### 3.4. Main results

Multivariable regression analyses were conducted to evaluate associations between pineapple consumption and study outcomes while accounting for potential confounders inherent to cohort data. Variables were entered into regression models based on established clinical relevance from prior literature (maternal age, parity, BMI, gestational age at delivery, and socioeconomic status) and baseline characteristics demonstrating a univariable association with outcomes at *P* < .10. All selected variables were retained in adjusted models irrespective of statistical significance to minimize residual confounding. Detailed regression outputs, including adjusted effect estimates, confidence intervals, and *P* values for all modeled outcomes, are presented in Table 3.

**Table 3:** Multivariable regression analysis of pineapple consumption and labor outcomes.

Outcome variable	Model type	Unadjusted estimate (95% CI)	*P* value	Adjusted estimate (95% CI)*	*P* value	Interpretation
Cervical ripening (Bishop score)	Linear regression	Mean difference: 0.8	<.001	Adjusted mean difference: 0.8 (0.5–1.1)	<.001	Higher Bishop score among pineapple consumers
Labor duration (h)	Linear regression	Mean difference: −1.7	<.001	Adjusted mean difference: −1.7 (−2.1 to −1.3)	<.001	Shorter labor duration associated with exposure
Spontaneous vaginal delivery	Logistic regression	OR not specified†	–	aOR 1.8 (1.4–2.3)	<.001	Increased likelihood of spontaneous vaginal delivery
Cesarean delivery	Logistic regression	OR not specified†	–	Inverse association implied (see mode of delivery outcome)	–	Lower CS rate among exposed group
Neonatal birth weight	Linear regression	No significant difference	NS	No significant association	NS	No exposure effect observed
Apgar score	Linear regression	No significant difference	NS	No significant association	NS	No exposure effect observed
NICU admission	Logistic regression	No significant difference	NS	No significant association	NS	No exposure effect observed

aOR = adjusted odds ratio, CI = confidence interval, NICU = neonatal intensive care unit, NS = not significant, OR = odds ratio.

* Adjusted for maternal age, parity, body mass index (BMI), gestational age at delivery, and socioeconomic status. Variables were selected based on clinical relevance and univariable association at *P* < .10.

† Unadjusted odds ratios not explicitly reported in narrative results; include if available from analysis output.

**Figure 3.**
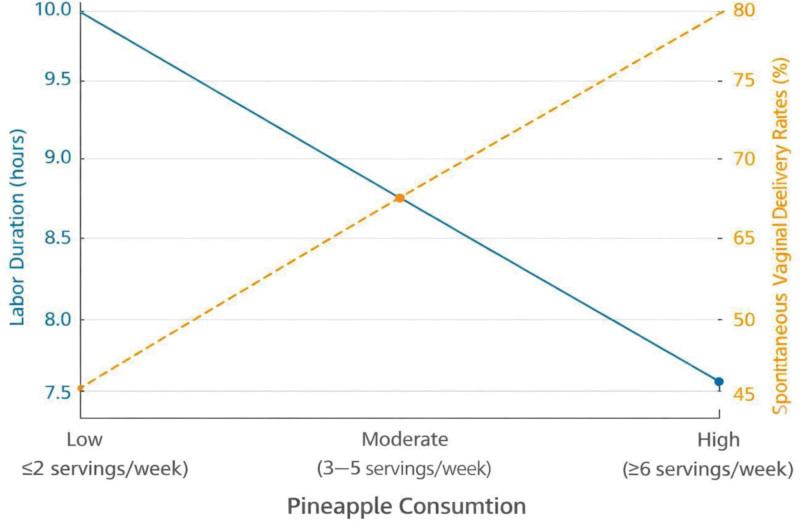
Dual-axis line graph illustrating labor duration and spontaneous vaginal delivery rates across pineapple consumption categories. Dose–response relationship between pineapple consumption and labor outcomes across predefined intake categories: low (≤2 servings/week), moderate (3–5 servings/week), and high (≥6 servings/week). Source: Authors’ Creations.

Unadjusted and adjusted analyses revealed significant associations between pineapple consumption and labor outcomes. First, cervical ripening: pineapple consumption was associated with a higher Bishop score (adjusted mean difference 0.8, 95% CI 0.5–1.1, *P* < .001). Linear regression models were used for continuous outcomes such as Bishop score and labor duration. Second, labor duration: pineapple consumption reduced labor duration by an average of 1.7 hours (adjusted mean difference −1.7, 95% CI −2.1 to −1.3, *P* < .001). Third, mode of delivery: women in the exposed group were more likely to have spontaneous vaginal delivery (adjusted odds ratio [aOR] 1.8, 95% CI 1.4–2.3, *P* < .001). Logistic regression was applied for binary outcomes including mode of delivery and neonatal endpoints. Categorization of pineapple consumption revealed a dose–response relationship: high consumers experienced the shortest labor durations and highest rates of spontaneous vaginal delivery (see Figs. 2 and 3).

### 3.5. Other analyses

**Figure 4.**
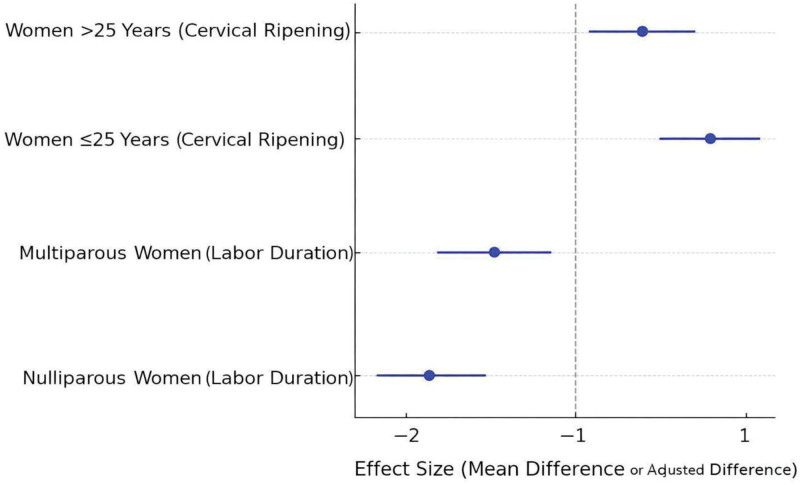
Forest plot showing subgroup effects of pineapple consumption on labor duration and cervical ripening.

Subgroup analyses indicated that nulliparous women benefited more significantly from pineapple consumption, with a 2-hour reduction in labor duration compared to a 1-hour decrease in multiparous women; women aged ≤25 years showed the greatest improvement in cervical ripening, with an adjusted mean difference of 1.2 (95% CI 0.8–1.6) (see Fig. 4). Sensitivity analyses confirmed the robustness of results across alternative exposure definitions and thresholds for labor outcomes. Missing data imputation did not alter the significance or direction of associations.

## 4. Discussion

Key findings from the present analysis indicate that pineapple consumption during pregnancy was associated with improved cervical ripening (higher Bishop scores), shorter labor duration, and an increased likelihood of spontaneous vaginal delivery, while neonatal outcomes remained comparable between exposure groups. These findings suggest a potential association with more favorable labor progression without detectable adverse neonatal effects within this cohort. Interpretation should be situated within the broader context of existing obstetric and reproductive health literature, emphasizing clinical relevance rather than mechanistic speculation.

Traditional methods, including mechanical and pharmacological interventions, have been extensively utilized to induce or augment labor when spontaneous onset is delayed or medically necessary.^[17]^ Compared with established obstetric benchmarks, the observed reduction in labor duration (approximately 1.7 hours) falls within a clinically meaningful range when contrasted with reported reductions achieved through nonpharmacologic adjuncts such as mobility protocols, supportive labor positioning, or dietary interventions described in obstetric literature.^[18]^ While direct equivalence cannot be assumed, these comparisons suggest that the magnitude of association observed in this study is potentially relevant from a clinical perspective.

Previous reproductive and fertility-focused studies investigating pineapple or bromelain exposure have largely examined implantation, inflammation modulation, and uterine receptivity rather than labor-related outcomes, with findings remaining inconsistent and primarily observational.^[19]^ The present findings extend this limited body of research by evaluating later-stage pregnancy outcomes, including cervical ripening, labor duration, and intrapartum progression, thereby expanding investigation beyond fertility-related endpoints.^[17]^

Biochemically, bromelain has been hypothesized to influence connective tissue remodeling through proteolytic activity; however, in the context of the present findings, mechanistic explanations remain speculative and should not be interpreted as evidence of causality.^[19]^ Variability in bromelain concentration due to fruit preparation, ripeness, and dietary absorption introduces uncertainty regarding biological plausibility when pineapple is consumed as part of a routine diet rather than as a standardized extract.^[20]^

Comparisons with prior observational studies reveal mixed findings. Some reports suggest associations between pineapple intake and shorter labor duration or improved cervical favorability, whereas others demonstrate no significant differences in labor progression or neonatal outcomes.^[21]^ Discrepancies across studies may reflect differences in exposure measurement (dietary frequency vs controlled supplementation), population characteristics, parity distribution, or adjustment for confounders.^[17]^ The use of multivariable regression in the present study strengthens inference compared with earlier descriptive reports but does not eliminate residual confounding.

Importantly, neonatal outcomes – including birth weight, Apgar scores, and NICU admission – did not differ significantly between groups. This aligns with several obstetric investigations examining dietary exposures during late pregnancy, which generally report minimal impact on neonatal parameters unless substantial nutritional deficiencies or excesses are present.^[22]^ The absence of adverse neonatal signals in this study provides preliminary reassurance regarding moderate pineapple consumption, although prospective confirmation is required.

Beyond mechanistic hypotheses, these observations also align with broader nonpharmacologic strategies aimed at optimizing labor progression. Obstetric literature increasingly recognizes dietary patterns, maternal activity, and supportive practices as potential modifiers of labor progression.^[23]^ The present results contribute to this growing body of evidence by evaluating a culturally relevant dietary exposure within a large cohort framework.

While pineapple consumption is culturally familiar in many settings, including Nigeria, the significance of this study lies primarily in its evaluation of measurable clinical outcomes rather than ethnographic observations. Accordingly, cultural practices are best understood as contextual factors influencing exposure rather than as explanatory mechanisms for the observed associations.^[24,25]^

A major limitation of existing literature is the scarcity of randomized controlled trials evaluating pineapple consumption clinical efficacy and safety during pregnancy. Most available studies are retrospective or rely on self-reported dietary habits, which may introduce recall bias and reduce the reliability of findings.^[26]^ Additionally, there is minimal consensus on the recommended dosage or timing of pineapple intake for optimal outcomes. Variability in study populations, including differences in maternal age, parity, and baseline health conditions, further complicates comparisons across studies.

Potential risks associated with pineapple consumption remain largely theoretical, and no increase in adverse obstetric or neonatal outcomes was observed in this cohort. Nevertheless, caution is warranted, particularly given variability in dietary exposure and absence of controlled dosing studies.^[27]^

### 4.1. Key results

This nationwide retrospective cohort study investigated the association between pineapple consumption during pregnancy and labor outcomes in Nigerian women. The results demonstrated significant differences between the exposed (pineapple consumers) and unexposed groups. First, cervical ripening: pineapple consumption was associated with improved cervical ripening, as evidenced by a higher mean Bishop score at admission (6.2 ± 1.3 vs 5.4 ± 1.5, *P* < .001). Second, abor duration: women in the exposed group experienced shorter labor durations (8.5 ± 2.1 hours) compared to the unexposed group (10.2 ± 2.4 hours, *P* < .001). Third, mode of delivery: the rate of spontaneous vaginal delivery was higher in the exposed group (65% vs 50%, *P* = .002), while cesarean section rates were lower (20% vs 30%, *P* = .005).Fourth, secondary outcomes: no significant differences were observed in neonatal birth weight, Apgar scores, or NICU admissions between the groups. Adjusted analyses supported these findings, indicating that pineapple consumption was associated with a higher Bishop score (adjusted mean difference 0.8, 95% CI 0.5–1.1, *P* < .001), reduced labor duration (−1.7 hours, 95% CI −2.1 to −1.3, *P* < .001), and increased odds of spontaneous vaginal delivery (aOR 1.8, 95% CI 1.4–2.3, *P* < .001).

### 4.2. Limitations

This study sheds light on important findings, but several limitations should be acknowledged. First, the reliance on self-reported dietary data may have introduced recall bias. However, this was mitigated through cross-referencing with antenatal records. Second, despite adjustments for maternal age, parity, and socioeconomic status, unmeasured confounders such as physical activity or other dietary habits could have influenced the results. Third,variability in the frequency and quantity of pineapple consumption may limit the precision of the exposure classification. Fourth,while this study captures a diverse Nigerian population, findings may not be generalizable to settings with different dietary patterns or healthcare systems. Finally, as a retrospective investigation, the study is limited in establishing definitive cause-and-effect relationships. These considerations underscore the need for future prospective studies to build on these insights.

### 4.3. Interpretation

The findings suggest that pineapple consumption during the 3rd trimester may positively influence cervical ripening and labor outcomes, aligning with traditional beliefs and preliminary biochemical evidence on bromelain effects. However, the retrospective design and potential biases necessitate cautious interpretation. The lack of significant differences in neonatal outcomes offers reassurance regarding the safety of moderate pineapple consumption during pregnancy. These results support the potential for culturally appropriate dietary interventions but highlight the need for prospective trials to validate findings and establish causality.

### 4.4. Generalisability

The study inclusion of participants from diverse socioeconomic and geographic backgrounds across Nigeria enhances its applicability to similar low-resource settings. However, generalisability to high-income countries or regions with distinct dietary patterns may be limited. Future studies should examine the impact of cultural and environmental factors on the observed associations.

## 5. Future directions

The findings from this study open several avenues for future research and exploration. First, additional prospective and randomized controlled trials must confirm the relationship between pineapple consumption and cervical ripening in diverse populations, considering socioeconomic, geographical, and cultural factors. Future studies could focus on identifying the active pineapple components responsible for potential benefits, including their mechanisms of action on the cervix and labor progression. Incorporating a wider range of variables, such as the timing, frequency, and quantity of pineapple consumption, could help establish clear guidelines for its use during pregnancy. Moreover, studying the impact of pineapple in combination with other dietary practices or supplements might provide insights into synergistic effects on maternal health and labor outcomes. It is also essential to explore the long-term effects of pineapple consumption on maternal and neonatal health, including potential adverse effects that may not be immediately apparent during labor. Future research should also examine how pineapple consumption might interact with preexisting medical conditions, such as gestational diabetes or hypertension, which are prevalent in many pregnant women. Another crucial aspect for future investigation is the role of cultural beliefs and practices related to food during pregnancy. Understanding how traditional and modern approaches can be integrated will provide a more holistic view of maternal care, allowing for tailored interventions that respect scientific evidence and cultural values. Finally, efforts to develop public health campaigns to educate women about safe and evidence-based dietary practices during pregnancy should be pursued. Collaboration with nutritionists, obstetricians, and midwives will be essential in fostering an environment where women can make informed decisions about their pregnancy to improve maternal and neonatal health outcomes.

## 6. Concluding remarks

This study aimed to explore the potential impact of pineapple consumption on cervical ripening and labor outcomes among pregnant women in Nigeria. While preliminary findings suggest that pineapple, due to its bromelain content, may have some influence on the ripening process and potentially shorten labor duration, the overall evidence remains inconclusive. The interplay of pineapple bioactive compounds with maternal physiology needs further investigation, mainly through large-scale randomized controlled trials that can provide more precise insights into the optimal dosage, timing, and safety of pineapple consumption during pregnancy. Despite the cultural relevance and widespread use of pineapple as a natural remedy for labor induction in Nigeria, the variability in individual responses, coupled with limited scientific evidence, calls for caution in endorsing its use as a standard clinical practice. Furthermore, while the fruit offers nutritional benefits, including vitamins and antioxidants supporting maternal and fetal health, excessive intake may pose potential risks, particularly concerning gastrointestinal discomfort or bleeding tendencies. To advance this area of research, future studies should aim to control for confounding variables such as diet, maternal health conditions, and socioeconomic factors. Additionally, a collaborative approach between medical professionals, traditional birth attendants, and nutritionists is essential to develop evidence-based guidelines that respect cultural practices while ensuring maternal and fetal safety.

## 7. Call to action

Given the findings of this study, further research must be conducted to understand better the potential role of pineapple consumption in cervical ripening and labor outcomes. We encourage researchers to undertake large-scale, randomized controlled trials to evaluate the efficacy, safety, and optimal consumption guidelines for pineapple during pregnancy. Additionally, healthcare professionals are urged to remain informed about the cultural practices surrounding pineapple consumption and its potential effects on labor. Obstetricians, midwives, and other maternal health practitioners must provide evidence-based advice to pregnant women while considering individual health conditions and preferences. Furthermore, a collaborative approach should be fostered between medical practitioners, nutritionists, and community health workers to educate the public about the nutritional benefits of pineapple and its potential effects on pregnancy. Such initiatives can contribute to a balanced understanding of traditional practices and modern medical guidelines, ensuring pregnant women receive the best care tailored to their needs. Ultimately, the goal is to create a safe, informed environment where conventional and traditional practices can coexist, promoting maternal and fetal health while addressing cultural preferences. The ongoing integration of scientific evidence into maternal healthcare practices will enhance the quality of care and empower women to make informed decisions about their pregnancy and labor experiences.

## Acknowledgments

The authors would like to express gratitude to all individuals and institutions that contributed to the completion of this paper. Their support, guidance, and encouragement throughout the research process are deeply appreciated.

## Author contributions

**Conceptualization:** Chukwuka Elendu, Dependable C. Amaechi, Tochi C. Elendu, Emmanuel C. Amaechi, Ijeoma D. Elendu.

**Data curation:** Chukwuka Elendu, Dependable C. Amaechi, Tochi C. Elendu, Emmanuel C. Amaechi, Ijeoma D. Elendu.

**Formal analysis:** Chukwuka Elendu, Dependable C. Amaechi, Tochi C. Elendu, Emmanuel C. Amaechi, Ijeoma D. Elendu.

**Funding acquisition:** Chukwuka Elendu, Dependable C. Amaechi, Tochi C. Elendu, Emmanuel C. Amaechi, Ijeoma D. Elendu.

**Investigation:** Chukwuka Elendu, Dependable C. Amaechi, Tochi C. Elendu, Emmanuel C. Amaechi, Ijeoma D. Elendu.

**Methodology:** Chukwuka Elendu, Dependable C. Amaechi, Tochi C. Elendu, Emmanuel C. Amaechi, Ijeoma D. Elendu.

**Project administration:** Chukwuka Elendu, Dependable C. Amaechi, Tochi C. Elendu, Emmanuel C. Amaechi, Ijeoma D. Elendu, Chinonso B. Okoro.

**Resources:** Chukwuka Elendu, Dependable C. Amaechi, Tochi C. Elendu, Emmanuel C. Amaechi, Ijeoma D. Elendu, Egbunu Emmanuel.

**Software:** Chukwuka Elendu, Dependable C. Amaechi, Tochi C. Elendu, Emmanuel C. Amaechi, Ijeoma D. Elendu, Joy Onomon, Egbunu Emmanuel, Peace Mordi, Chinonso B. Okoro.

**Supervision:** Chukwuka Elendu, Dependable C. Amaechi, Tochi C. Elendu, Emmanuel C. Amaechi, Ijeoma D. Elendu, Joy Onomon, Egbunu Emmanuel, Peace Mordi.

**Validation:** Chukwuka Elendu, Dependable C. Amaechi, Tochi C. Elendu, Emmanuel C. Amaechi, Ijeoma D. Elendu, Joy Onomon, Egbunu Emmanuel, Peace Mordi, Chinonso B. Okoro.

**Visualization:** Chukwuka Elendu, Dependable C. Amaechi, Tochi C. Elendu, Emmanuel C. Amaechi, Ijeoma D. Elendu, Joy Onomon, Egbunu Emmanuel, Peace Mordi, Chinonso B. Okoro.

**Writing – original draft:** Chukwuka Elendu, Dependable C. Amaechi, Tochi C. Elendu, Emmanuel C. Amaechi, Ijeoma D. Elendu, Joy Onomon, Egbunu Emmanuel, Peace Mordi, Chinonso B. Okoro.

**Writing – review & editing:** Chukwuka Elendu, Dependable C. Amaechi, Tochi C. Elendu, Emmanuel C. Amaechi, Ijeoma D. Elendu, Joy Onomon, Egbunu Emmanuel, Peace Mordi, Chinonso B. Okoro.
